# Microstructure and Mechanical Behavior of Quaternary Eutectic α+θ+Q+Si Clusters in As-Cast Al-Mg-Si-Cu Alloys

**DOI:** 10.3390/ma16186091

**Published:** 2023-09-06

**Authors:** Kai Li, Yan Yu, Qiang Lu, Yuanfei Li, Qiao Yan, Xinyue Lan, Liya Li, Baishan Chen, Min Song

**Affiliations:** 1 State Key Laboratory of Powder Metallurgy, Central South University, Changsha 410083, China; leking@csu.edu.cn (K.L.); 15116455244@163.com (Y.Y.); luqiangsdly@163.com (Q.L.); 223312132@csu.edu.cn (Y.L.); 213311028@csu.edu.cn (Q.Y.); 2023007@aust.edu.cn (X.L.); msong@csu.edu.cn (M.S.); 2 Institute for Advanced Study, Central South University, Changsha 410083, China

**Keywords:** homogenization, transmission electron microscopy, eutectic clusters, AlCuMgSi clusters, intermetallics, dendritic

## Abstract

Cu additions notably strengthen Al-Mg-Si and Al-Si-Mg alloys due to the dense precipitation of quaternary nano precipitates during ageing. However, the chemical evolution and mechanical behaviors of the quaternary micro-scale Q constituent phase occurring in cast and homogenized states have rarely been studied. Meanwhile, there exists a type of AlCuMgSi cluster in the cast state, which has been regarded as Q particles. The accurate identification of phase constituents is the basis for the future design of alloys with better performance. In our work, this type of cluster was revealed to consist of α-Al, θ-Al_2_Cu, Q, and Si phases through micro-to-atomic scale studies using scanning and transmission electron microscopes. The skeleton of the dendrite was θ phase. The second phases in the dendritic eutectic cluster dissolved quickly during a 4 h homogenization at 550 °C. The Q phase was found to effectively absorb the Fe impurities during casting and homogenization. As a result, the formation of other harmful Fe-rich intermetallics was suppressed. These Q constituent particles were observed to break into separate pieces in an intermediately brittle manner when compressed in situ in a scanning electron microscope. These findings provide insights into the thermodynamic modeling of the Al-Mg-Si-Cu system and alloy design.

## 1. Introduction

Al alloys have been called the energy bank for the future [1], in this era of depleting resources and energy, serving as the second-largest industrial alloy system (after steel [2]), widely used in transportation, green-building, and packaging all over the world. Al alloy products can be recycled with only 5% of the energy cost of producing aluminum from bauxite ore [3]. The excellent corrosion resistance, good formability, and high strength/weight ratio of 6xxx series age-hardenable Al-Mg-Si(-Cu) alloys [4] are especially interesting to the automobile and aircraft industries, where products’ durability, safety properties, manufacturing costs, and fuel economy are of critical importance. Typical products include rolled auto body panels [5], forged auto chassis parts [6], and extruded parts. Recently, making cars at low cost with a unibody casting of Al alloys is becoming more and more popular, especially among electric car manufacturers. The microstructural control of Al alloys, starting from the control at the micro-scale during casting, is of critical importance to these applications.

The Q phase is a key phase in Al alloys, including not only 6xxx series Cu-added Al-Mg-Si alloys [7], but also 4xxx series Cu-added Al-Si-Mg alloys [8,9] and 2xxx series Si-added Al-Cu-Mg alloys [10], existing as as-cast constituents [8,10] and nano-precipitates [9,11]. Abundant research has been conducted to clarify the precipitation behaviors of Al-Mg-Si(-Cu) alloys during age hardening, especially paint bake hardening, a typical heat treatment process for auto body panels [5], thus guiding the design of new generations of alloys. In these alloys, Cu additions have been found to induce the formation of Cu-containing nano-precipitates, such as Q [2,12,13,14] in the over-aged state, its precursor Q′ [7,15,16,17,18], C, S, L, QP [19], and QC [20,21], as well as β″-Al_2_Mg_5_Si_4_ [22] with Cu substitutions [23] in the peak-aged state; meanwhile, the alloys are hardened to higher levels compared to Cu-free alloys with the same Mg and Si contents [12]. Similar results have also been reported in Al-Si-Cu-Mg alloys heat-treated after casting [24,25].

The crystal structure model for the bulky Q phase (Al_4_Cu_2_Mg_8_Si_7_, space group $P\bar{6}$) was first constructed by Arnsberg et al. [26] through X-ray diffraction (XRD) studies of an annealed single-phase sample; the parameters a = 1.039 nm and c = 0.402 nm were determined.

The compositional and structural features of Q nano-precipitates in aged Al-Mg-Si-Cu alloys have been intensively studied over the past 20 years. The crystal structure of rod-like Q nano-precipitates (sometimes termed Q′) has been studied mainly via transmission electron microscopy (TEM). Compared to the Al_4_Cu_2_Mg_8_Si_7_ structure [26], it has the same space group and slightly different parameters (a = 1.03 nm, c = 0.405 nm) according to the electron diffraction studies conducted by Sagalowicz et al. [27]. In their work, the stoichiometry of Al_5_Cu_2_Mg_8_Si_6_ was suggested. The stoichiometry was recently updated to be Al_6_Cu_2_Mg_6_Si_7.2_ by Wenner et al., based on atomic resolution elemental mapping results that were obtained from scanning transmission electron microscopy–energy dispersive X-ray (STEM-EDX) signals [22].

In contrast, few studies have focused on the compositional characteristics, morphological features, and mechanical properties of the micro-scale quaternary constituents, such as the Q phase, in cast or homogenized Al alloys containing Mg, Si, and Cu [28], and thus, inconsistencies exist. In the excellent work by Löffler, Rettenmayr, and coworkers [29], directional solidification was used to prepare the millimeter scale Q phase, and its composition was determined to be around Al_3_Cu_2_Mg_9_Si_7_ (in at. %) using energy dispersive X-ray spectrometry (EDXS). Wu et al. [30] reported on the spherical cluster-like and needle-like morphologies of the micro-scale quaternary AlCuMgSi particles in the as-cast samples and furnace-cooled samples (after homogenization) of an Al-0.9%Mg-0.9%Si-0.6%Mn-0.42%Cu-0.1%Ce-0.2%Fe (weight fraction) alloy, respectively, using scanning electron microscopy (SEM) and EDXS. It is interesting that the two morphologies correspond to two compositions; the needle-like one is not too far away from the compositions previously reported, while the spherical one has a very strange atomic ratio of Cu:Mg:Si = 2:3.7:8.5. They inferred that the spherical cluster was the product of the binary eutectic reaction L → α-Al + AlCuMgSi. Such eutectic clusters, appearing in either a spherical shape or an irregular shape, with varying contrasts inside when viewed through SEM, have been widely reported [7,11,13,31,32,33,34], as summarized in Table 1. Usually, these AlCuMgSi clusters are regarded as Q particles and not as clusters, although their compositions vary from each other. What are the fine structures and phase constituents inside these AlCuMgSi clusters? What are the differences between them and the bulky AlCuMgSi particles? These are questions to answer.

The accurate identification of phase constituents in cast and homogenized states is the basis of future design of alloys with better performance, and thus systematically clarifying the micro-scale quaternary clusters and bulky quaternary particles in the quaternary Al-Mg-Si-Cu alloy system is of critical importance. In this work, the phase constituents in three Al-Mg-Si-Cu alloys were investigated by means of SEM, TEM, and EDXS. The implications of the obtained results were discussed in relation to future design of high-strength, high-toughness Al-Mg-Si-Cu alloys.

## 2. Experimental Section

### 2.1. Materials and Preparation

The compositions of the alloys studied in this work are listed in Table 2. All compositions in this paper are given in weight fraction unless specified. The alloys were cast in an induction furnace into cylindrical ingots with a diameter of 17 mm and a height of 80 mm, and then air-cooled. Alloy #1 was cast from industrial Al ingots (purity 99.7%), high-purity Mg ingots (99.9%), and Al-20.2%Si and Al-49.5%Cu master alloys. The actual composition of alloy #1 was determined to be Al-1.03%Mg-1.18%Si-0.63%Cu-0.03%Fe, using inductively coupled plasma atomic emission spectrometry (ICP-AES). The detected Fe impurities were mainly from the industrial Al ingots, as all other raw materials had impurities less than 0.1%. More details of the casting process have been described in another paper [11]. The cylindrical cast ingots were cut normal to the long axis into small disks with electro-discharge machining. Some disks of alloy #1 were later homogenized at 550 °C for 4 h and water-quenched.

Alloys 2 and 3 were similarly cast, except that high-purity (99.999%) Al ingots and high-purity (99.9%) iron powder were used. The actual compositions of alloys 2 and 3 are believed to be very close to the designed ones, as all processes that might cause compositional deviation were strictly controlled. Alloy #1 was designed for industrial purposes. Alloys #2 and #3 are model alloys for comparison.

### 2.2. Characterizations

The transverse cross-sections of the disks were observed with an FEI Helios Nano Lab 650 or an FEI Quanta 250 SEM instrument. The samples were not coated or treated before SEM observation because of the high electrical conductivity of Al alloys. Some SEM samples were etched in a solution of 85 vol.% phosphoric acid, 5 vol.% nitric acid, and 10 vol.% acetic acid, for 5–10 min at room temperature. Thin foil specimens for TEM observations were cut from the transverse cross-sections of the disks, ground, and thinned using twin-jet electro-polishing in a solution of 30 vol.% nitric acid and 70 vol.% methanol at about −30 °C. Low-magnification TEM and high-resolution transmission electron microscopy (HRTEM) observations were performed in an image-corrected FEI Titan G2 60-300 TEM instrument operated at 300 kV. Focused ion beam (FIB) preparation of plane-view TEM sample from the surface of a sample of alloy #1 was performed with a Thermo Fisher Helios 5UC dual beam FIB/SEM instrument. Atomic resolution high angle annular dark field scanning transmission electron microscopy (HAADF-STEM) was performed on this TEM sample with a double-corrected Thermo Fisher Spectra 300 TEM instrument operated at 60 kV.

In situ nano-indentation tests were performed with a Hysitron PI-87 SEM PicoIndenter mounted in an FEI Helios Nanolab 600 FIB/SEM instrument (FIB: focused ion beam). The Berkovich indenter was used in the displacement-control mode. The indentation depth of 800 nm was used for a protruding Q phase particle. The loading speed was 100 nm/s.

## 3. Results

### 3.1. The Quaternary Eutectic Cluster Found in All Three Alloys

**Figure 1:**
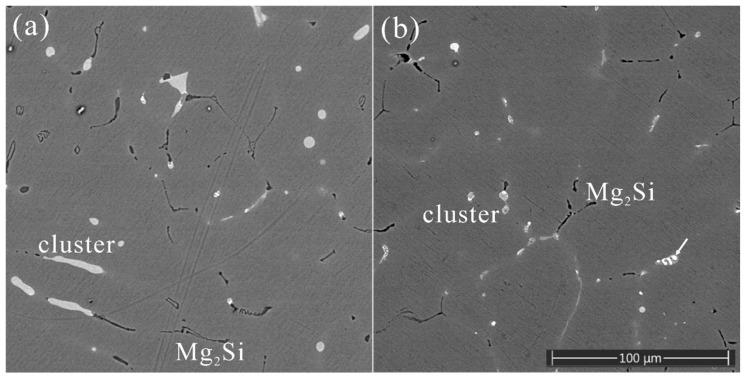
Backscattered electron (BSE) images of the as-cast microstructures of (**a**) alloy #2 and (**b**) alloy #3. The brightest, second brightest, and darkest contrasts are from θ (see the white arrow) particles, the AlMgSiCu cluster, and β-Mg_2_Si particles.

Along with Mg_2_Si phase, a type of quaternary eutectic cluster containing Al, Mg, Si, and Cu elements was found in as-cast microstructures of all three alloys. Our previous work on the as-cast microstructure of alloy #1 revealed the existence of such a eutectic cluster [11]. The eutectic cluster had a rod-like morphology with a dendritic internal structure. The as-cast microstructures of alloy #2 and alloy #3, as shown in Figure 1, were compared with that of alloy #1 to reveal the influence of minor variations in chemical composition on the formation of the quaternary eutectic cluster. Alloy #2 has an as-cast microstructure comprised of quaternary eutectic clusters and β-Mg_2_Si particles, which appear white and dark in the backscattered electron (BSE) images in Figure 1a, respectively. The microstructure of as-cast alloy #3 is very similar except for that there are large bright particles inside the eutectic clusters, as shown in Figure 1b.

**Figure 2:**
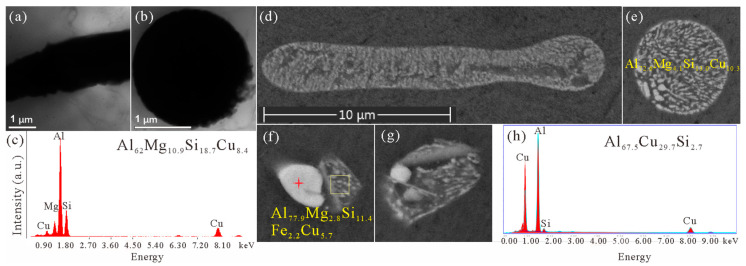
(**a**–**c**) Morphological and compositional characteristics of eutectic clusters in the as-cast microstructure of alloy #1. The two particles in TEM images (**a**,**b**) are both enriched in Al, Mg, Si, and Cu at very similar levels, and the EDX spectrum for the particle in (**b**) is shown in (**c**). The quantified contents of elements in atomic fractions are also given in (**c**). (**d**–**h**) Morphologies and compositions (in at. %) of eutectic clusters in the as-cast microstructures of (**d**,**e**) alloy #2 and (**f**–**h**) alloy #3, respectively. The EDX spectrum in (**h**) is from the position marked by a red cross in (**f**). The four BSE images (**d**–**g**) share the same scale bar.

The TEM images of the as-cast microstructure of alloy #1, as shown in Figure 2a,b, also reveal the rod-like morphology of the quaternary eutectic clusters. Their diameters are measured to be around 2 μm (from over 10 clusters). The typical EDX spectrum in Figure 2c reveals the enrichment of Al, Mg, Si, and Cu in the cluster.

In high-magnification SEM images of alloy #2, such as in Figure 2d,e, the rod-like morphology and dendritic eutectic structures inside the rods were again observed, which were no different from those observed in alloy #1 [11]. In alloy #3, with 0.05 wt.% Fe addition, the eutectic clusters contained considerable Fe content (2.2 wt.%), as shown in Figure 2f. The large particles formed inside the clusters, which appeared brighter than other parts of the clusters, were confirmed as θ-Al_2_Cu by EDX studies, as shown in Figure 2h. It is worth mentioning that EDX studies on micron-scale particles in SEM are largely affected by the neighboring matrix or potential particles beneath the studied ones, due to the thick sample used. Consequently, the compositions obtained by EDX studies in SEM in this work will not be discussed in a quantitative way. Despite this, the compositions of different clusters, as listed in Figure 2c,e,f, vary largely. Such a strange compositional variation implies that the cluster contains more than one phase other than the α-Al matrix phase.

Traditional sample preparation methods such as twin-jet electro-polishing or ion milling have difficulty producing the preferred TEM sample with uniform thickness and minimal overlapping of different dendrite arms (as thin as 50 nm in diameter). As a result, the TEM images in Figure 2a,b have lost the contrast variation that they should have, confirming this trouble in overlapping. On the other hand, although the SEM images in Figure 2e–g have almost reached the optimal resolution that an ordinary FEG SEM can have, no third type of contrast emerges. Therefore, FIB was applied to prepare a uniformly thin TEM sample, and a double-aberration-corrected TEM instrument was used for fine structure observations down to the atomic scale in the following part.

### 3.2. FIB/SEM + TEM Identification of the Phases inside the Eutectic Clusters: α-Al+θ+Q+Si

**Figure 3:**
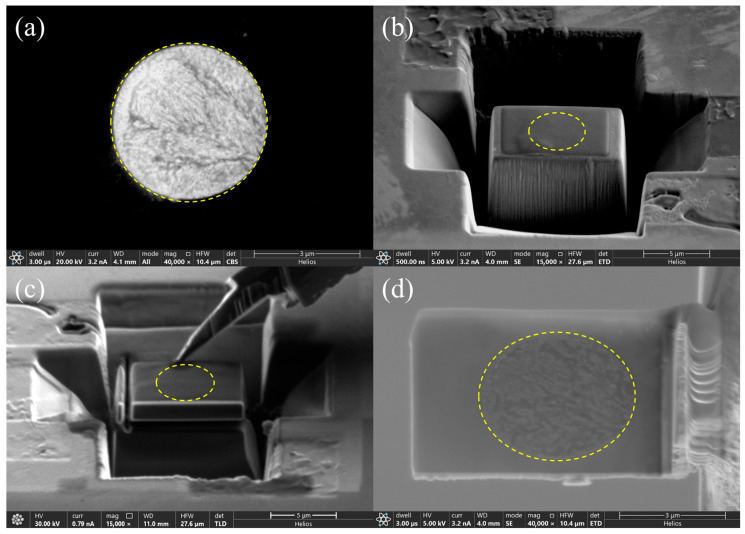
FIB preparation processes of a plane-view TEM sample from the surface of an SEM sample of alloy #1. (**a**) An end-on eutectic cluster was observed, and (**b**) Pt was deposited on this area and nearby areas were milled with the ion beam. (**c**) Then, this surface area was welded onto an Omniprobe nanomanipulator using Pt deposition and separated from the substrate sample by the ion beam. (**d**) Finally, this extracted thin sample was moved and welded onto a Cu grid, separated from the Omniprobe, and further thinned with the ion beam. The yellow dashed circle denotes the edge of the cluster.

**Figure 4:**
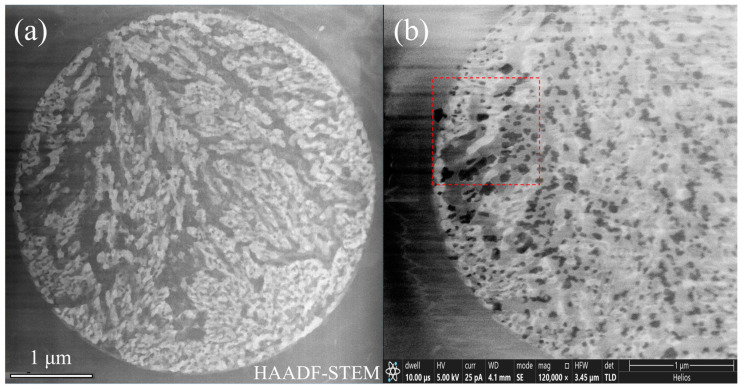
Fine structures inside the TEM sample prepared with FIB. (**a**) HAADF-STEM image obtained in TEM. (**b**) Secondary electron image obtained in SEM.

A plane-view TEM sample containing a eutectic cluster on the surface of alloy #1 was prepared, step-by-step, as shown in Figure 3. In this thin TEM sample, the cluster displayed four types of contrast (bright, grey, dark grey, and dark) in both HAADF-STEM mode in TEM and SE mode in SEM, as shown in Figure 4a,b, respectively.

**Figure 5:**
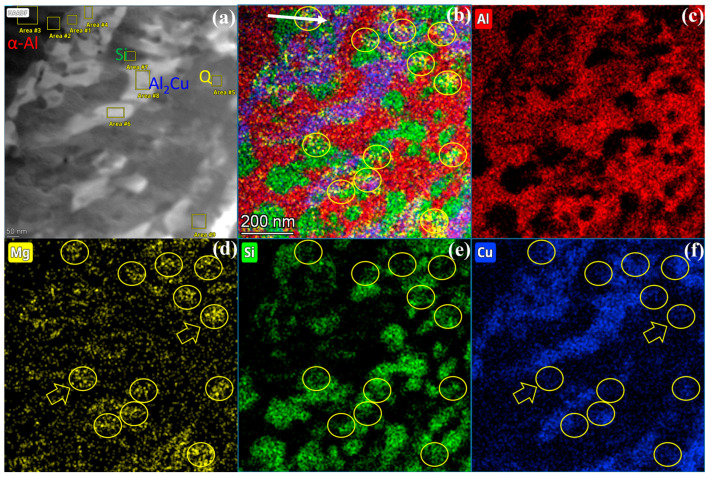
Compositional features of various phases inside the eutectic cluster. (**a**) High-magnification HAADF-STEM image, (**b**) mixed, and (**c**–**f**) individual elemental distribution according to EDX mapping results of the same area. The yellow circles and yellow arrows denote Mg_2_Si particles.

**Figure 6:**
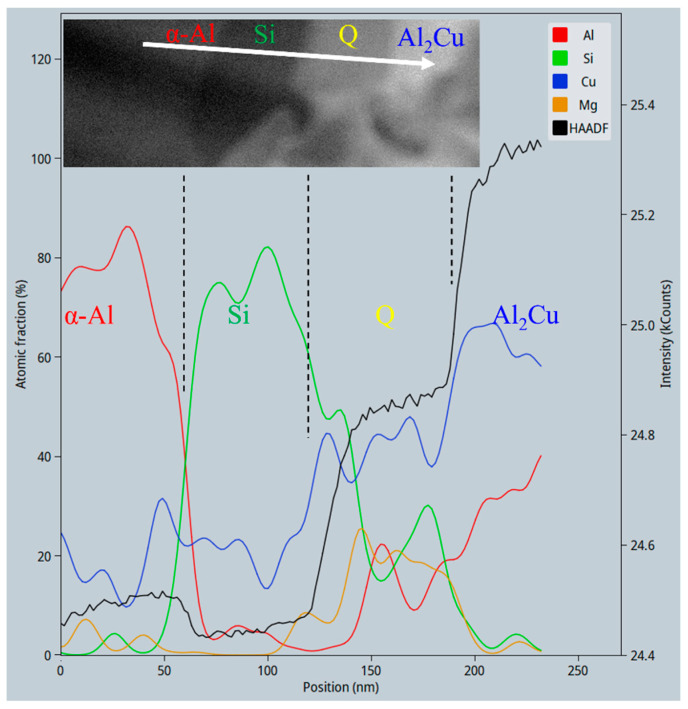
Line profile of EDX signals along the white arrow in Figure 5b. The y axis on the right denotes the intensity in the HAADF-STEM image. A magnified HAADF view of the area along the same white arrow is also inserted.

The area in the red dashed frame in Figure 4b was further analyzed with HAADF-STEM and EDX in high magnifications, as shown in Figure 5 and Figure 6 (and also Supplementary Figure S1). The four phases, from the brightest to the darkest, as denoted in the inset image and the black curve in Figure 5, correspond to Al_2_Cu, Q (probably Al_4_Cu_2_Mg_8_Si_7_), α-Al, and Si, respectively. This can be explained by the mass-thickness contrast characteristics of HAADF-STEM images. Al_2_Cu has the highest Cu content (Z = 29), while the Q phase has much less Cu. Si (Z = 14) atoms are heavier than Al (Z = 13) atoms, but the Si phase has a substantially lower density of atoms than α-Al. The Al_2_Cu phase constitutes the main skeleton of the dendritic cluster.

It should be noted that the EDX signals from the phases were mixed with strong background Cu signals, and even in the α-Al and Si phases there was around 13–15 at. % Cu (see the blue curve in Figure 6 and Table 3). Such background Cu signals might be from the EDX signals from the Cu grid supporting the FIB-prepared TEM lamellar sample, or from the Cu atoms that were sprayed from the Cu grid onto the sample. Therefore, the typical compositions of Q and Al_2_Cu listed in Table 3 were subtracted by the minimal artifact Cu content, retrieving calibrated compositions of Al_10.3_Cu_4.3_Mg_8.0_Si_7.9_ and Al_1.09_Cu, respectively. There are still some residual background Cu signals in both phases, and the Al content in the Q phase was influenced by the EDX signals from the neighboring α-Al matrix. In a word, unfortunately, these compositions can only be interpreted in a semi-quantitative way.

**Figure 7:**
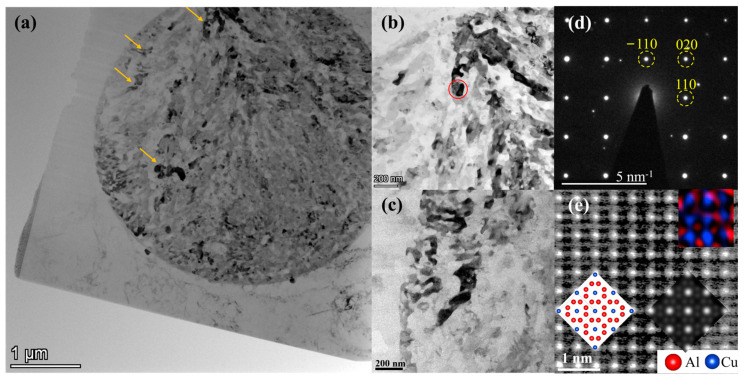
Orientational and structural studies of the θ-Al_2_Cu dendrites inside the eutectic cluster. (**a**) Bright field image of the whole cluster. (**b**,**c**) Enlarged view of some areas. (**d**) SAED pattern of the area in red circle in (**b**). (**e**) Atomic resolution HAADF-STEM image of θ-Al_2_Cu, in comparison with the standard structure, the simulated HAADF-STEM image, and EDX elemental maps inserted in the lower left, lower right, and upper right corners. Zone axes of figures (**b**–**e**) are all parallel to [001] of θ-Al_2_Cu. Yellow arrows in (**a**) indicate the areas intensively studied in this work.

The TEM bright field and atomic resolution HAADF-STEM images shown in Figure 7 confirm that the crystal structure of the white branches in Figure 4 and Figure 5 is a tetragonal θ-Al_2_Cu structure, which has a I4/mcm space group and lattice parameters a = b = 6.067 Å, c = 4.877 Å according to the work of Meetsma et al. [35]. As shown in the bright field images in Figure 7a–c, when a branch is tilted to a zone axis, some other branches share the same zone axis and similarly display dark contrast. In Figure 7d, this zone axis was identified as the [001] axis of the θ-Al_2_Cu structure using selected area electron diffraction (SAED). This implies that different branches inside the dendrite cluster were grown on the same nucleus. It should be noted that the TEM sample surface was normal to the long axis of the cylindric cast ingot, which underwent a positive temperature gradient from bottom to top during solidification [11]. In other words, the [001] axis of these θ-Al_2_Cu branches was parallel to the temperature gradient. However, it is currently not known if all branches belong to approximately the same orientation or not. In other words, it is not known by how many degrees the [001] axis of each of the other branches deviates from the long axis of the cluster. In the future, a full study of the orientations of all branches using scanning precession electron diffraction or 4D-STEM would be interesting.

The atomic resolution HAADF-STEM image of θ-Al_2_Cu along this axis, as shown in Figure 7e, corresponded with the structure and the simulated HAADF-STEM image inserted, especially in the distribution of Cu atoms. HAADF-STEM image simulation was performed in QSTEM software 2.51 [36] using defocus −10 nm, thickness 9.6 nm, and collection angle range 64–200 mrad. The distribution of Al and Cu using EDX mapping was also inserted in the same figure. It should be noted that the FIB-prepared sample might be apparently thicker than 9.6 nm, and this limits the spatial resolution of HAADF-STEM and EDX images. In any case, the distribution of Cu columns revealed by both HAADF-STEM and EDX was consistent with the standard θ-Al_2_Cu structure, and the Al columns were found around the Cu columns.

In a word, using various FIB/SEM and TEM characterizations, the phases inside the eutectic clusters were identified as α-Al, θ-Al_2_Cu, Q, and Si. This probably implies a eutectic reaction L → α+θ+Q+Si during the solidification of the melt.

### 3.3. The Fast Dissolution Behavior of the Eutectic Clusters during Homogenization

**Figure 8:**
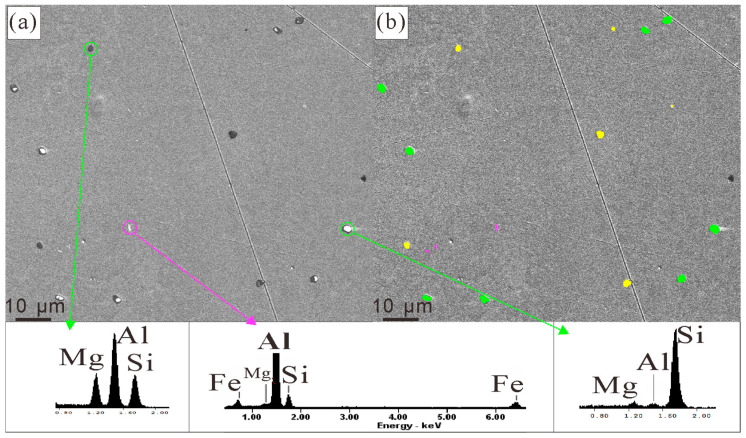
Identification and measurement of volume fractions of the particles in the homogenized microstructure of alloy #1: (**a**) is the secondary electron (SE) image and (**b**) is the processed one. Typical EDX results are shown as insets. The particles marked in (**b**) in green are Si particles, those marked in yellow are Mg_2_Si particles, and those in purple are ternary AlFeSi particles.

In the homogenized microstructure of alloy #1, three major types of phases, β-Mg_2_Si, Si, and β-Al_9_Fe_2_Si_2_, were found through EDX in SEM, as shown in Figure 8. There were equiaxed β-Mg_2_Si and Si particles with dimensions of 1~3 μm. The particle types of β and Si were confirmed using EDX, as shown in the insets of Figure 8. The particles identified as Si particles had, in fact, a very high Si content and negligible Mg content, whereas those identified as β-Mg_2_Si particles had notable Mg and Si content. The volume fractions of the β-Mg_2_Si, Si, and β-Al_9_Fe_2_Si_2_ particles measured in the SEM image (of a 200 μm × 200 μm area) were, respectively, 0.17 ± 0.01%, 0.15 ± 0.01%, and 0.011 ± 0.002%.

One of the predominant second phases, θ-Al_2_Cu, was fully dissolved during a homogenization heat treatment at 550 °C for 4 h and was rarely seen in the homogenized microstructure. Such a homogenization heat treatment is far shorter than similar treatments in other Al-Mg-Si-Cu alloys. For example, in Samuel’s work, bulky θ-Al_2_Cu phase was not fully dissolved during a heat treatment of a cast Al-Si-Cu-Fe alloy at 540 °C for 24 h, while the (Al + Al_2_Cu) eutectic almost fully dissolved after 8 h [37]. In other words, the dissolution of dendritic θ-Al_2_Cu phase in the current alloy is far more effective than the bulky θ-Al_2_Cu phase in other Al-Mg-Si-Cu alloys, primarily due to its larger specific surface area associated with the dendritic morphology. The fast dissolution of θ-Al_2_Cu phase is beneficial for solute homogeneity in real-world alloys. When the added Cu has all been dissolved into the α-Al solid solution, the dense precipitation of Cu-containing β″ precipitates [23] and other precipitates will be promoted during artificial ageing. Meanwhile, the size and number density of micro θ-Al_2_Cu particles, which are generally not beneficial for the strength and ductility, will be reduced. This is critical for improving the strength and other properties of the alloys.

### 3.4. Residual Fine Q Particles Enriched with Iron in the Homogenized Microstructure

**Figure 9:**
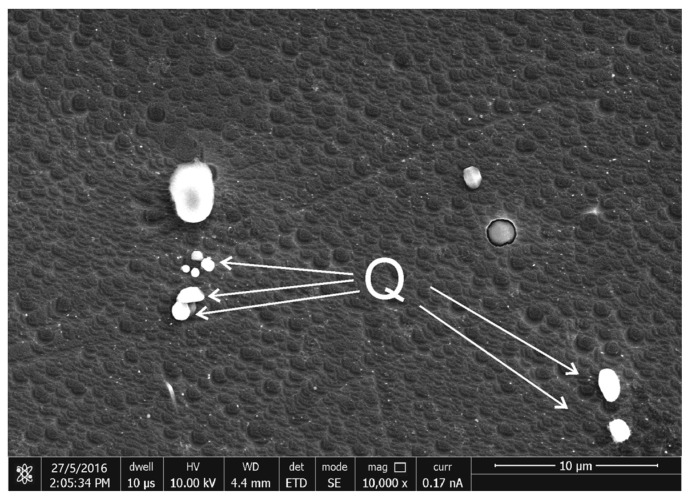
SE images of the etched surfaces of the homogenized sample of alloy #1.

The Q phase is a key phase in various Al alloys, existing as micro-scale as-cast constituents and nano-precipitates. Currently there are Al_4_Cu_2_Mg_8_Si_7_, Al_5_Cu_2_Mg_8_Si_6_, and Al_6_Cu_2_Mg_6_Si_7.2_ models for the Q phase. Fine Q particles have also been found in the homogenized microstructure of alloy #1, probably as remnants of the eutectic clusters in the as-cast microstructure. Observations after chemical etching were performed to reflect their true morphological features. They were in nearly spherical morphologies and diameters ranging from 0.4 μm to 1 μm, as shown in Figure 9.

**Figure 10:**
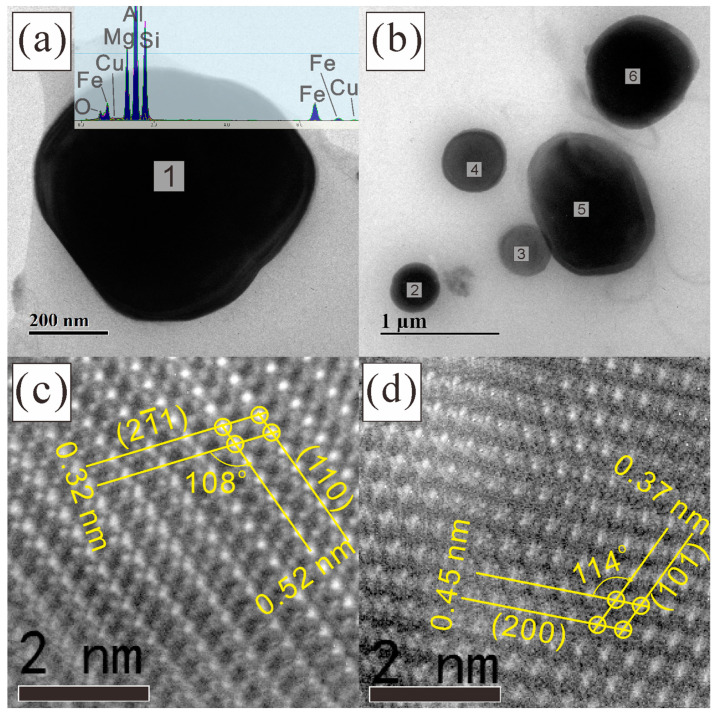
Observations of the Q particles in the homogenized alloy #1 using (**a**,**b**) TEM and (**c**,**d**) HRTEM. The lattice of particle 1 was observed along (**c**) [$\bar{1}$13]_Q_ and (**d**) [010]_Q_.

**Figure 11:**
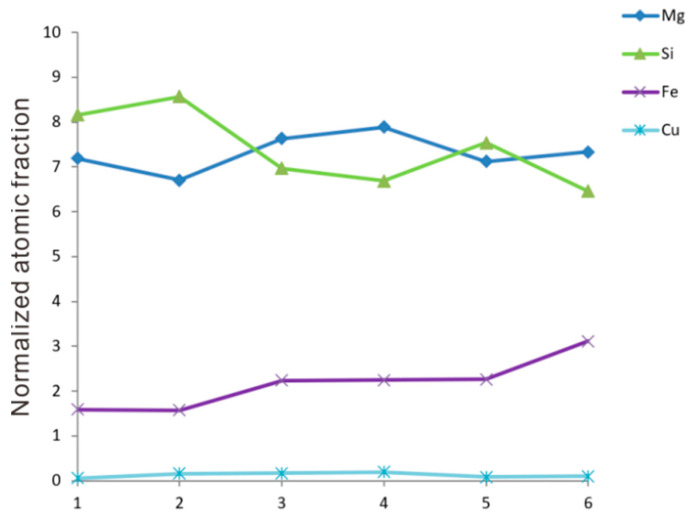
Compositions of the six Q particles in Figure 10 obtained using EDX studies. The Mg, Si, Fe, and Cu content (in atomic fraction) was normalized to a total amount of 17.

The composition and crystal structures of these fine Q particles were investigated with HRTEM, as shown in Figure 10. It should be noted that the Cu content decreased substantially to an ignorable level and seemed to be replaced by Fe, as can be seen in the typical EDX spectrum inserted in Figure 10a and the Q compositions listed in Figure 11. This is not an occasional phenomenon but holds true for every Q particle found in the homogenized microstructure. Specifically, if one presumes an Al_4_Cu_2_Mg_8_Si_7_ stoichiometry according to the work of Arnberg et al. [26], the ratio *x*_(Cu+Fe)_:*x*_Mg_:*x*_Si_ should be close to 2:8:7, and this fits the statistic results in Figure 11 well, although some scatter indeed exists. More precise compositional determination using wavelength-dispersive spectrometry (WDS) in an electron probe microanalyzer (EPMA) is difficult because these Q particles are too small. In contrast, in the Q phase in the as-cast microstructure of the current alloy, the EDX peaks for Fe at about 6.4 keV are always negligible.

### 3.5. The Brittle Nature of Q Phase Particles

The elastic modulus of the Q phase was measured as 117.76 ± 12.51 GPa by Chen et al. via nano indentation tests, and it was smaller than that of most other intermetallics in the as-cast microstructure of an Al-14.61%Si-4.6%Cu-3.26%Ni-0.78%Mg-0.65%Fe-0.04%Mn alloy [38]. Although the Q phase is already known to be soft, whether it is brittle or ductile was not clear from their work, due to the limited indentation depth (less than 400 nm) and the large flat sample surface. Such missing information is crucial for evaluating the probability of cracking during the deformation of the alloy, e.g., during deformation processing and during service, and is of critical importance for the design of Cu-containing Al-Mg-Si or Al-Si-Mg alloys, or Mg-added Al-Cu-Si alloys [28].

**Figure 12:**
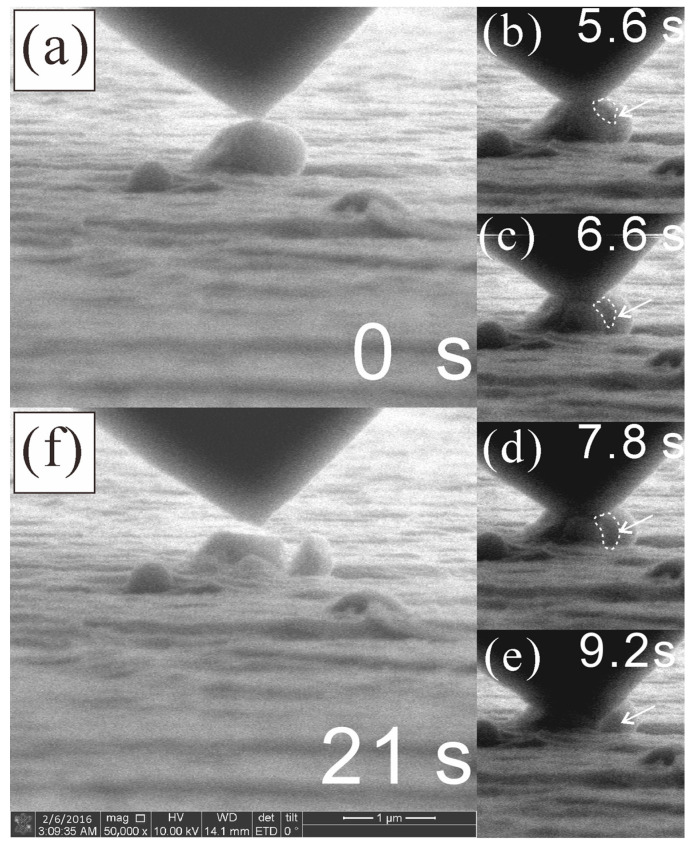
Frames captured in the in situ compressive experiment in SEM (SE mode) on an etched sample in the homogenized state: (**a**–**f**) denote different stages from the load–unload–lift-up procedure, with a total duration of 21 s. The arrows and dashed curves indicate the evolution of a crack in the particle.

The brittle nature of fine Q phase particles has been revealed using the in situ compression test in SEM, as shown in Figure 12. The frames captured from the video reveal the process of indenter contact (Figure 12a) → crack generation (Figure 12b) → crack propagation (Figure 12c,d) → final rupture of the whole particle (Figure 12e). Since the contrast from the crack is indeed weak here, dashed curves were plotted as a guide for the eyes; meanwhile, the raw video has been uploaded as a supplementary file. The areas in the dashed curves are confirmed as weaker in intensity than adjacent areas in image processing software. In any case, after unloading and lifting the indenter, the particle was found to be ruptured into several separate pieces. This is solid evidence for the brittleness of the Q phase.

**Figure 13:**
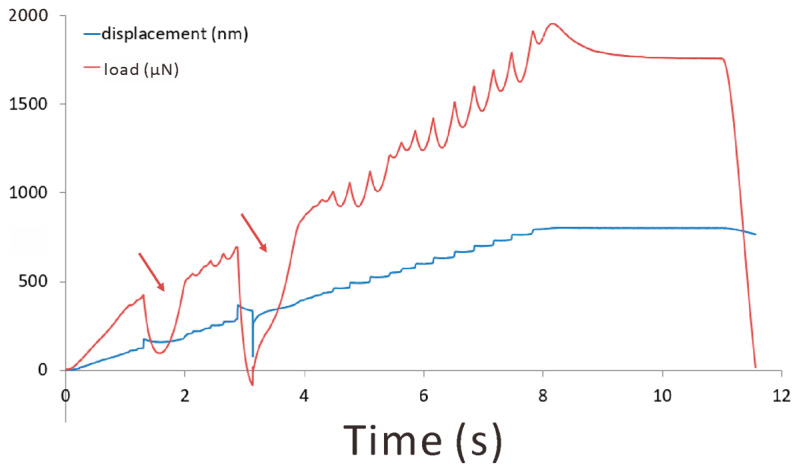
Evolution of load and displacement with time during in situ mechanical tests of the Q phase particle. The red arrows indicate the load drop events.

The brittle fracture during the indentation of the Q particle was also well reflected in the curves of evolution of load and displacement with time during loading (see Figure 13). As the red arrows in the figure denote, the load abruptly dropped for a while and then recovered during deformation, corresponding to a turbulence of the displacement curve. The load drop can be explained by the mechanism of crack generation → propagation → lower load required for the proceeding of deformation → cracks blunted. Such behaviors are very similar to those of some fly ash particles tested in the work of Ding et al. [39]. Those deformed fly ash particles also exhibited cracks and load-drop features. They were classified as less brittle than those particles that fractured into pieces all of a sudden after the smooth evolution of the load–displacement curve. In reference to their work, the Q particles studied in this work were classified as intermediately brittle, i.e., there is a process of micro-crack evolution before the final brittle fracture.

## 4. Discussion

### 4.1. The Significance of the Fast Dissolution of the θ-Al_2_Cu Phase in the Dendritic Eutectic Cluster

Compared to Mg_2_Si and Si phases, the θ-Al_2_Cu phase is more difficult to dissolve due to the relatively slow diffusion of Cu in Al [40]. Therefore, the fast dissolution of θ-Al_2_Cu is one of the key points in the homogenization heat treatment of these Al-Mg-Si-Cu alloys. In addition, the fast dissolution of Cu-containing phases, e.g., θ-Al_2_Cu and Q phases, is of critical importance and strongly affects the properties. In Samuel’s work on cast Al-Si-Cu alloys [37], tensile strength and elongation properties (of test bars heat treated at 480–515 °C for 2–24 h) showed a linear increase when plotted against the amount of dissolved copper in the matrix. On the other hand, if the alloys are further aged artificially, the full dissolution of these phases is the guarantee of a large driving force for nano-precipitation. Otherwise, undissolved θ-Al_2_Cu and Q may initiate cracks during the deformation and degrade the mechanical properties.

### 4.2. The Effect of Iron-Absorbing Q Constituents on Phase Transformations in Al-Mg-Si-Cu Alloys

The Q particles contained no Fe content in either the as-cast state of alloy #1 (with about 0.03 wt.% Fe impurities) and alloy #2 (with almost no Fe). In alloy #3, which was added with 0.05 wt.% Fe, as shown in Figure 2f, there was substantial Fe content (nearly equal to the Mg content) incorporated in the eutectic clusters. In the homogenized microstructure of alloy #1, nearly spherical Q particles were found, containing substantial Fe content but negligible Cu content. It is thus inferred that the Q particles in both the as-cast microstructure and homogenized microstructure, may absorb Fe, and a higher Fe content in the alloy or a longer diffusion time at high temperatures is beneficial for the incorporation of Fe in Q phase. This observation suggests that the Fe content plays a role in the formation and composition of the Q phase.

In all three alloys studied in this work, including alloy #3, which had 0.05% Fe artificially added, there were very few elongated β-Al_9_Fe_2_Si_2_ particles [41] (cross-section of a flake), as shown in Figure 8a. In alloys #2 and #3, which were prepared from high-purity raw materials and relatively clean processes, all element contents were strictly controlled, and detected amounts closely adhered to the nominal compositions. The added Fe content of 0.05% was a reasonable simulation of the Fe impurity content in industrial casting processes. Thus, it can be inferred that the formation of brittle β-Al_9_Fe_2_Si_2_ compounds can be efficiently suppressed in Al-Mg-Si-Cu alloys containing the Q phase.

The finding of suppressing the formation of AlFeSi compounds by forming an iron-absorbing Q constituent is significant for designing high-strength, high-toughness Al-Mg-Si-Cu alloys. In Al alloys, AlFeSi compounds mainly include β-Al_9_Fe_2_Si_2_ phase [41] and α-Al_8_Fe_2_Si phase [42], which occur in flake-like and Chinese-script-like morphologies, respectively. Due to the brittle nature and the large stress concentration, β flakes are considered to be extremely detrimental to the mechanical properties of aluminum alloys. They will lead to the cracking of aluminum alloys [43] during deformation in processing or in use, resulting in poor surface quality and poor mechanical properties.

### 4.3. The Effects of the Intermediately Brittle Nature of Q

The potential effects of the intermediately brittle Q particles on the mechanical properties of Al-Mg-Si-Cu alloys are manifold. Indeed, these particles might break during deformation and degrade the mechanical properties of as-cast Al-Mg-Si-Cu alloys. In the homogenization heat treatment, the particles were greatly refined and dissolved due to the Q phase not being stable at high temperatures above 430 °C, according to the calculated phase equilibria of the nominal compositions of alloys #1–3 at various temperatures shown in Supplementary Figures S2 and S3. The subsequent rolling process and solution heat treatment would further enhance the refinement and dissolution of Q particles. This is consistent with the work of Wang et al. on Al-Mg-Si-Cu alloys, in which a short solution heat treatment for only 2 min at 555 °C effectively reduced the amount of the Q phase [44]. In this stage, the Fe impurities released by such dissolution barely resulted in the formation of Fe-rich intermetallics due to the slow diffusion of Fe, while the Mg, Si, and Cu solutes released were beneficial for the dense precipitation of nano-phases such as β″ in the subsequent ageing process, finally leading to a high strength of Al-Mg-Si-Cu alloys.

More accurate mechanical properties of the Q phase will be determined in the future. It is necessary to conduct further experiments using advanced testing techniques such as nanoindentation, atomic force microscopy, or mechanical testing at varying strain rates and temperatures. These experiments would provide more accurate measurements of mechanical properties such as the hardness, Young’s modulus, and yield strength of the Q phase.

### 4.4. Suggestions for the Future Refinement of Thermodynamic Databases on Al Alloys

The precise prediction of solidification paths and phase equilibria is of critical importance for alloy design. This paper is not aimed at pointing out mistakes in currently popular commercial databases, but to suggest some areas for refinement in the future, as stated in the following paragraphs:

(1) The solubility of Fe in the Q phase should be incorporated in its thermodynamic model in the future for a more precise prediction of phase equilibria, as well as solidification paths, since Fe almost completely took the Cu sites in the Q phase in the homogenized Al-Mg-Si-Cu alloys with Fe impurities (see Figure 11) and partly took the Cu sites in Q in the as-cast state (see Figure 2f). None of the current thermodynamic databases for multi-component multi-phase Al alloys contain an iron-free thermodynamic model for the Q phase. This is crucial for alloy design, as the precise prediction of solidification paths and phase equilibria is essential for the microstructural control of Al alloys during casting processes.
(2) The standard chemical formula(s) of the Q phase in as-cast and homogenized states needs reliable experimental investigation in industrial alloys. Currently, there are Al_4_Cu_2_Mg_8_Si_7_, Al_5_Cu_2_Mg_8_Si_6_, and Al_6_Cu_2_Mg_6_Si_7.2_ models. Atomic-scale studies of the structure, composition, and atomic occupancy of the micro or sub-micro Q particles in industrial Al-Mg-Si-Cu alloys are lacking. This is of key importance for establishing the thermodynamic model of the Q phase occurring in these states.
(3) During the thermodynamic assessment of systems containing Al, Mg, Si, and Cu, the widely appearing quaternary eutectic cluster with α-Al, θ-Al_2_Cu, Q, and Si phases might have been regarded as a Q phase or α-Al + Q eutectic cluster (see Table 1). This needs to be double checked.

This study on the quaternary eutectic α+θ+Q+Si cluster in as-cast Al-Mg-Si-Cu alloys provides valuable insights into the microstructural features and their implications for alloy design and applications. The identification and characterization of the Q phase, along with its interaction with other phases, contribute to our understanding of the alloy’s microstructure and its influence on mechanical properties.

## 5. Conclusions

In this work, comprehensive studies through SEM, TEM, EDX, HRTEM, HAADF-STEM, and in situ nano-indentation tests in SEM were applied to the characterization of a type of eutectic cluster in Al-Mg-Si-Cu alloys, with or without trace Fe impurities. The chemical evolution and mechanical behaviors of the quaternary Q constituent particles were also studied. The following conclusions were obtained:

(1) The eutectic cluster has a dendritic structure containing α-Al, θ-Al_2_Cu, Q, and Si phases, with the θ-Al_2_Cu phase as the main skeleton.
(2) The dendritic structure facilitates the fast dissolution of phases in the eutectic cluster, especially the θ-Al_2_Cu phase, which is difficult to dissolve in bulk morphology. After 4 h of homogenization at 550 °C, the eutectic clusters disappeared and only some fine remnant Q particles were left.
(3) The remnant Q constituent particles in the homogenized microstructure of the Al-Mg-Si-Cu alloy containing Fe were found to be enriched with notable Fe content, which almost took all the Cu sites in the Q structure. Higher Fe content in the alloy or a longer diffusion time at high temperatures is beneficial for the incorporation of Fe in the Q phase. The absorption of the Fe impurity element by the Q phase efficiently suppressed the formation of other Fe-rich intermetallics such as the detrimental β-Al_9_Fe_2_Si_2_ phase. This information is important for improving mechanical properties through alloy design.
(4) The Q particle broke into separate pieces in intermediately brittle behavior during an in situ nano-indentation test in SEM. This feature is indeed harmful for the mechanical properties of as-cast alloys, but can be beneficial in other states. The fragmented Q phase during the deformation processes can then be efficiently dissolved in high temperatures, promoting the dense precipitation of β″ phases during ageing and improving strengthening effects.
(5) For the precise prediction of solidification paths and phase equilibria, it is important to incorporate the Fe element in the thermodynamic model of the Q phase, and to clarify the standard chemical formula(s) of the Q phase in as-cast and homogenized states based on reliable experimental investigation in industrial alloys. The quaternary eutectic α+θ+Q+Si clusters might have been regarded as Q phase or α + Q clusters, and these should be double checked.

These findings contribute to a better understanding of the phase transformations and have implications for the design and processing of high-performance 6000 series Al-Mg-Si-Cu alloys and other series of Al alloys containing Mg, Si, and Cu.

## Figures and Tables

**Table 1:** Compositional and morphological features of various AlCuMgSi clusters and particles [7,11,31,32,34]. In this table and throughout the paper, compositions are expressed in weight percentage unless specified.

Alloy and State	Morphology	Composition	Comments
As-cast 2214 [7]	honeycomb	not provided	identified as Q phase by the authors
Al-1.15Mg-0.92Si-1.99Cu [7,34]	intertwined structure	not provided	identified as Q phase by the authors
Al-2.081Cu-1.165Mg-0.981Ni-0.865Fe-0.281Zr-0.118Si-0.088Ti [31]	round-shaped particles with non-uniform contrast in SEM	Al-32.95Cu-4.65Mg-1.92Si-0.20Fe-1.20Ni (at. %)	identified as Al_2_Cu phase by the authors
Al-0.84Mg-0.96Si-0.53Cu-0.16Mn [32]	round-shaped plates with non-uniform contrast in SEM	Al-29.31Mg-27.76Si-10.43Cu (at. %)	identified as Q phase by the authors
Al-0.80Mg-1.08Si-0.54Cu-0.11Mn-0.21Sb [32]	round-shaped plates with non-uniform contrast in SEM	Al-17.53Mg-44.67Si-7.20Cu-1.68Mn (at. %)	identified as Q phase by the authors
Al-1.0Mg-1.1Si-0.65Cu [11]	rods with a dendrite like internal structure	with an atomic ratio Cu:Mg:Si = 2:2.6:4.5	identified as Q phase by the authors

**Table 2:** Nominal compositions of the 3 alloys used in this work (wt.%).

Alloy	Mg	Si	Cu	Fe	Others	Al
1	1.0	1.1	0.65	0	<0.03	balance
2	1.0	1.0	0.65	0	<0.03	balance
3	1.0	1.0	0.65	0.05	<0.03	balance

**Table 3:** Determined compositions (in at. %) of various phases (marked as areas #1–9) in Figure 5a.

Area	1	2	3	4	5	6	7	8	9
Mg	26.44	1.40	1.29	2.02	25.65	2.32	0.56	0.65	16.31
Al	20.22	2.59	82.28	38.63	32.86	46.30	3.54	45.13	35.11
Si	21.25	82.87	1.01	2.13	20.20	2.85	80.83	1.28	26.12
Cu	32.09	13.14	15.41	57.21	21.29	48.52	15.07	52.95	22.46
Phase	Q	Si	α-Al	θ-Al_2_Cu	Q	θ-Al_2_Cu	Si	θ-Al_2_Cu	Q

## Data Availability

Data are available upon request.

## Supplementary Materials

The following supporting information can be downloaded at: https://www.mdpi.com/article/10.3390/ma16186091/s1, Figure S1: Compositional features of various phases inside the eutectic cluster. (a) HAADF-STEM image obtained in TEM. (b,g) High-magnification HAADF-STEM image. (c–f) Individual elemental distribution according to EDX mapping results of the area (b). (h) Mixed and (i–l) individual elemental distribution according to EDX mapping results of the area (g); Figure S2: Calculated phase equilibria of the nominal composition of alloy #1 at various temperatures from 170 °C to 550 °C. The thermodynamic database used was TCAL5. A reaction occurred at 430 °C, above which the Q phase cannot stably exist; Figure S3: Calculated phase equilibria of alloy #2 at various temperatures. The x-axis represents temperature in K from 300 K (27 °C) to 823 K (550 °C), and the y-axis represents the volume fraction of the solid phase. Video S1: In situ compression test in SEM.
