# Supplementary figures and images for: Microstructure and Mechanical Behavior of Quaternary Eutectic α+θ+Q+Si Clusters in As-Cast Al-Mg-Si-Cu Alloys
Source: Materials (Basel). 2023 Sep 6;16(18):6091. doi: 10.3390/ma16186091 (PMC10532811; doi:10.3390/ma16186091)

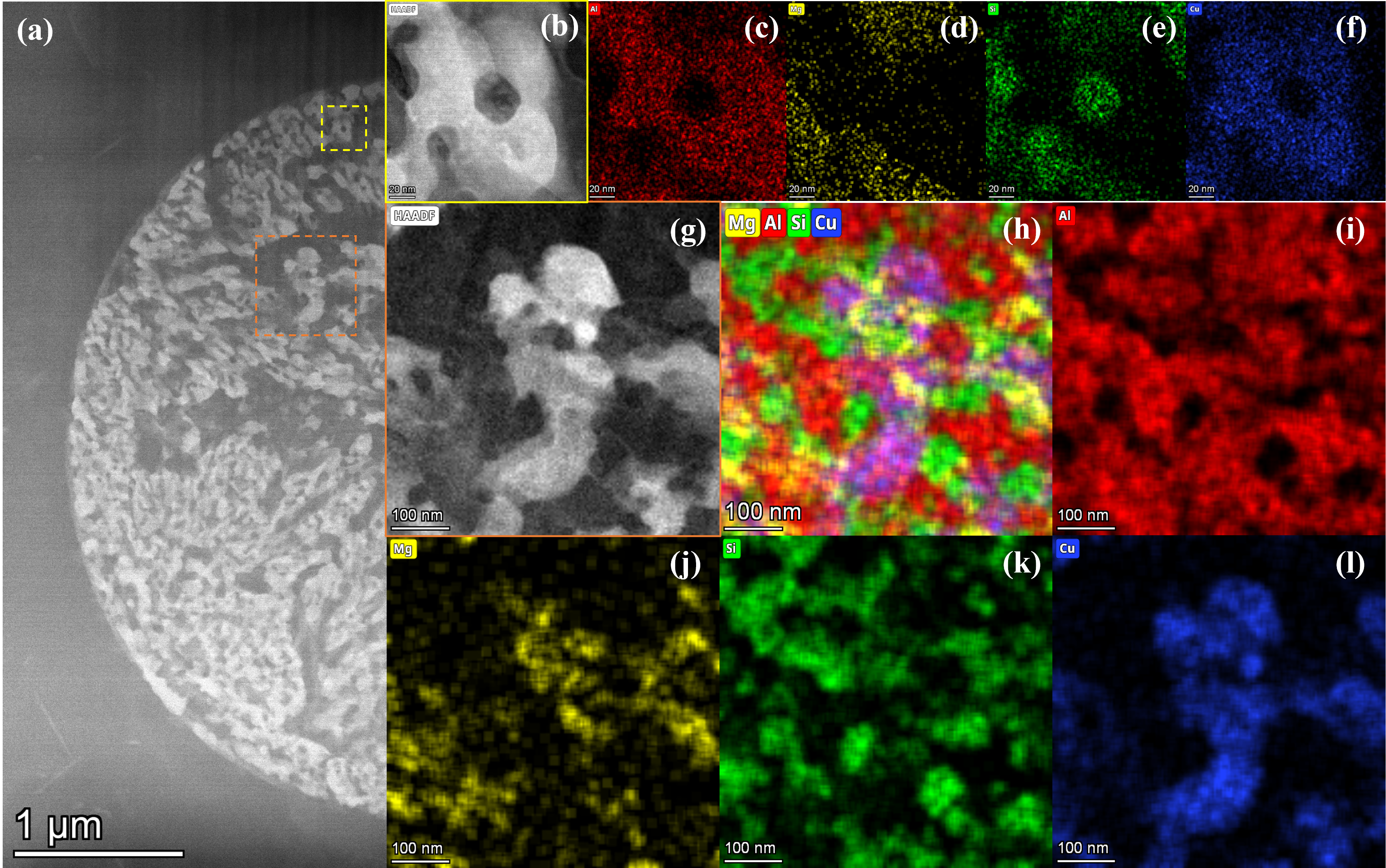

Supplement: Supplementary file 1 [file materials-16-06091-s001.zip › Figure S1.tif]

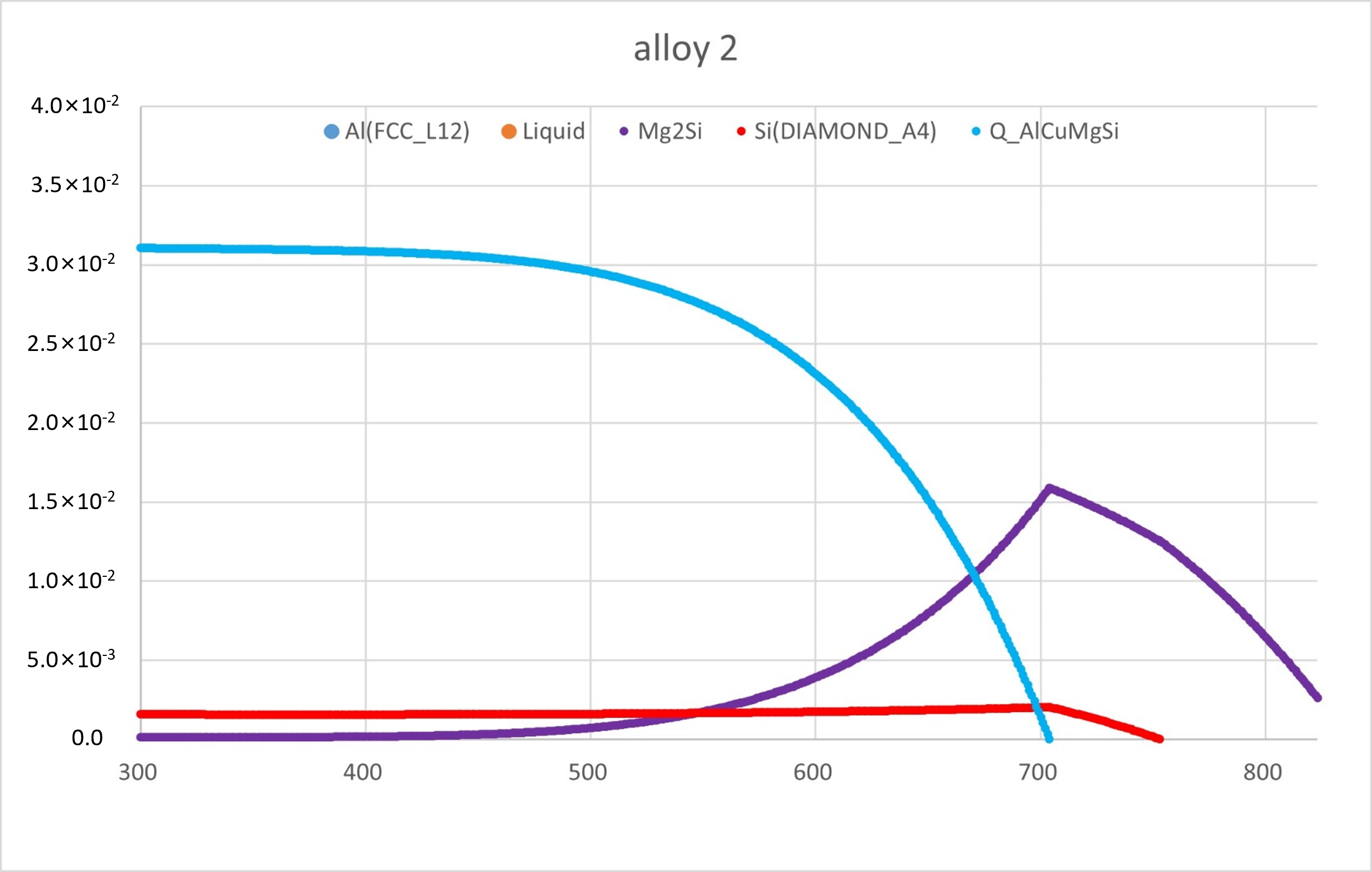

Supplement: Supplementary file 1 [file materials-16-06091-s001.zip › Figure S3.tif]
